# Effects of foam rolling and static stretching on ankle dorsiflexion and jumping ability: a randomized controlled trial

**DOI:** 10.5114/biolsport.2025.150042

**Published:** 2025-05-08

**Authors:** Antonino Patti, Ewan Thomas, Valerio Giustino, Carlo Rossi, Antonio Paoli, Patrik Drid, Antonio Palma, Antonino Bianco

**Affiliations:** 1 Sport and Exercise Sciences Research Unit, Department of Psychology, Educational Science and Human Movement, University of Palermo, 90144 Palermo, Italy; 2 Department of Biomedical Sciences, University of Padua, 35131 Padua, Italy; 3 Faculty of Sport and Physical Education, University of Novi Sad, 21000 Novi Sad, Serbia

**Keywords:** Myofascial release, Flexibility, Range of motion, Strength, Stability, Jump performance

## Abstract

This study aimed to assess the impact of a 5-week intervention that combined foam rolling (FR) and static stretching (SS) on ankle dorsiflexion range of motion (ROM). Furthermore, the study evaluated the influence of the intervention on leaping ability utilizing the counter-movement jump test (CMJ). Random assignment was used to allocate fifty-one amateur sports enthusiasts to either the foam rolling + static stretching (FR-SS) group (n = 22; age: 19.3 ± 6.87 years; height: 171 ± 7.91 cm; weight: 66.7 ± 9.52 kg) or the static stretching (SS) group (n = 29; age: 18.5 ± 4.60 years; height: 171 ± 11.5 cm; weight: 68.3 ± 16 kg). A baseline assessment (T0) and an assessment following the 5-week intervention (T1) were carried out. An inertial sensor was used to quantify the ankle joint ROM, and an optical detecting system was used to measure the CMJ performance. Significant differences were found between T0 and T1 in the FR-SS group. Significant group-by-time interaction effects (F = 14.44; p < 0.001, η^2^p = 0.128) and between-subject effects (F = 53.5; p < 0.001, η^2^p = 0.353) were found. Significant enhancements in CMJ performance (p < 0.01) were noted after the intervention in the FF-SS group. A similar trend was not observed in the SS group. Our findings suggest that a 5-week intervention combining FR with SS leads to greater improvements on ROM and angular velocity of the ankle joint, and on CMJ performance compared to SS alone. If further research confirms these results, FR and SS could be widely used in sports and rehabilitation.

## INTRODUCTION

An ankle sprain is a common occurrence that can occur at any age [1]. The aetiology of this condition is the elongation of the fibres or collagen in the ankle ligaments, resulting in partial or complete disruption of the fibres [2–4]. Individuals with a history of ankle sprain often experience chronic ankle difficulties, including chronic discomfort, muscle weakness, and symptoms associated with chronic ankle instability [4].

Chronic ankle instability (CAI) is a pathological disorder that often occurs following a lateral ankle sprain (LAS) [5]. The literature indicates that recurrent incidents of LAS resulting from CAI may decrease a person’s physical activity levels and health-related quality of life and could also lead to degenerative ankle joint conditions [6]. Furthermore, individuals who experience ankle sprains report strength and postural deficits [7]. Nevertheless, the physiological reason behind the impairments in strength following an ankle sprain remains poorly understood. In 2008, Hertel J. proposed a hypothesis on the cause of muscular dysfunction [8]. The potential explanation for this could be alterations in the excitability of the alpha motor neuron pool resulting from muscular inhibition caused by arthrogenic factors [8]. People with ankle sprains and/or CAI experience a decrease in their ability to sense and respond to stimuli and difficulties in controlling their muscles, leading to a lack of stability during movement [8].

One aspect contributing to CAI is decreased range of motion (ROM) in dorsiflexion [9]. Decreasing this range has a negative effect on stability during functional movements since a sufficient range of dorsiflexion is essential to achieve a stable position of the ankle joint [10, 11]. Consequently, patients with CAI may experience recurrent LAS and episodes of the ankle giving way due to this restricted dorsiflexion [12]. The significance of the dorsiflexion range has been acknowledged in the literature [13]. Pope R. et al. established that the extent to which the foot can be flexed upward (dorsiflexion range) can be used to predict the likelihood of experiencing fractures in the tibia or foot, tibial periostitis, ankle sprains, Achilles tendonitis, and anterior tibial compartment syndrome [13]. In the final analysis, increasing the ROM in dorsiflexion decreases the likelihood of getting ankle sprains and other major injuries in the lower limbs [14].

Enhancing the ROM is typically accomplished through several stretching techniques in fitness and rehabilitation programs to improve performance and minimize the likelihood of injury [15]. Several studies have evaluated acute changes in ROM after static stretching (SS). Many studies have shown varying degrees of dorsiflexion ROM improvements immediately after stretching [16–18]. Smith et al. hypothesized that the acute modification in the dorsiflexion ROM could be affected by both the duration and intensity of stretching [19]. Previous studies have shown that foam rolling (FR) can improve the ROM without diminishing muscular strength or performance [20, 21]. However, the long-term effects of medium-length interventions have been mixed.

In 2018, Hodgson et al. analyzed a four-week foam rolling (FR) intervention, comparing its effects when administered three times weekly, four times weekly, and against a control group [22]. The authors described a roller massage training that comprised four 30-second sets focusing on the quadriceps and hamstrings of the dominant limb, executed unilaterally [22]. This training did not show significant changes, indicating that the rolling-induced acute improvements reported in other studies may be transient [22]. In 2024, Kasahara et al. demonstrated that the FR intervention effectively altered the ROM and tissue hardness, irrespective of the rolling duration [21]. The authors described three intervention conditions composed of fast (1 rolling/2 s, 30-rep. × 3 sets, 90 rep.), medium (1 rolling/6 s, 10-rep. × 3 sets, 30 rep.), and slow speed (1 rolling/12 s, 5-rep. × 3 sets, 15 rep.) [21]. However, these effects were shown to remain for a length of 20–60 minutes [21]. On the other hand, Smith et al. reported a significant increase in ROM after a 6-week FR program, with results comparable to those of a SS intervention [19]. Similarly, Kiyono et al. demonstrated a significant increase in ROM after a 5-week FR intervention [23]. Recent studies confirm the effectiveness of FR on ROM but indicate that interventions longer than 4 weeks are needed for lasting gains, with evidence suggesting that responses may be specific to certain muscles or joints [24, 25].

Hence, the aim of this study was to evaluate the influence of a 5-week intervention that combines FR and SS on the ROM of ankle dorsiflexion and, consequently, on its stability.

Furthermore, several studies have investigated the correlation between ankle dorsiflexion and jumping ability and agility [26, 27]. While some studies have demonstrated enhanced performance with increased dorsiflexion, others have not, indicating that dorsiflexion can indeed alter muscle activation but does not necessarily lead to improved performance [26]. Jumping performance improvements can be linked to ankle dorsiflexion, which increases torque production in the triceps surae muscle group [28]. Some data indicate that dorsiflexion affects muscle activation patterns [29]. The literature indicates that lengthening an activated muscle leads to a temporary increase in strength during the lengthening and a sustained improvement in residual strength afterward [28]. However, Bourgit et al. also indicated that the dorsiflexion could reorganize the motor pattern [29].

Considering this, we have also evaluated the jumping ability with a counter-movement jump test.

## MATERIALS AND METHODS

### Experimental set-up

**FIG 1:**
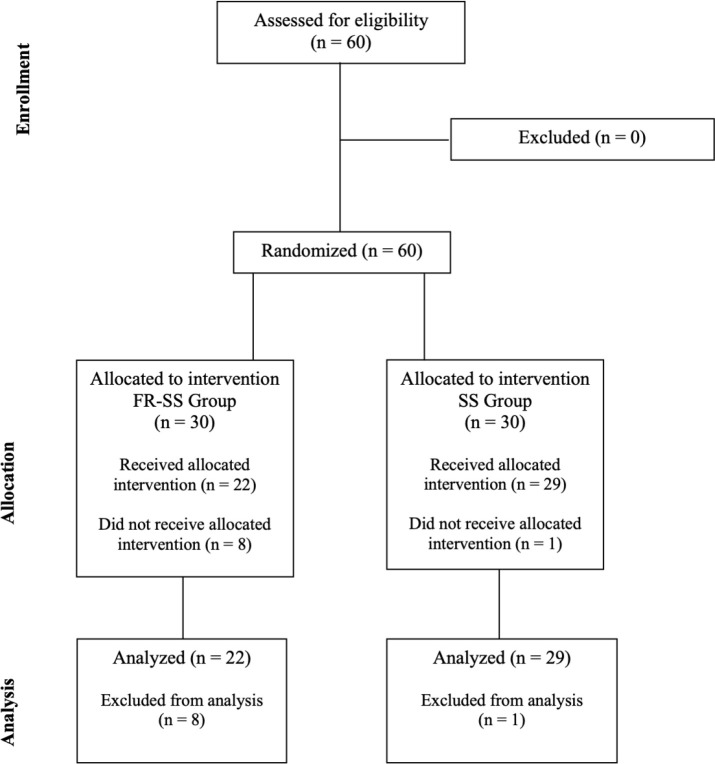
The CONSORT diagram shows the flow of participants through each stage of the randomized trial.

A randomized controlled design was employed to investigate the impact of FR on the posterior of the lower legs and foot-rolling on the plantar sole. The procedures are outlined in Figure 1. The participants were randomized in the foam roller + static stretching (FR-SS) and only static stretching (SS) groups using an online randomization tool (https://www.randomizer.org/). In detail, participants were randomly allocated to FR-SS and SS with a 1:1 ratio.

The assessment sessions occurred before the interventions (T0) and after 5 weeks (T1) by the same researcher, who was blinded to the intervention received by each participant and who was unaware of the study’s purpose. The ROM measures were conducted without wearing shoes and in a random sequence in both the dominant and non-dominant legs, as determined by the Side Preference Inventory (Coren, 1993). Both legs were evaluated to explain the reported alterations. In the same lines, the CMJ test was also administered randomly to all enrolled participants.

The participants were exposed to the following two interventions: a) an intervention on four days for week, where the SS group performed a SS program targeting the ankle flexors (the gastrocnemius, soleus, plantaris, popliteus, flexor hallucis longus, and flexor digitorum longus); b) the FR-SS group added a FR program for the gastrocnemius and soleus, and a foot roller program for the popliteus, flexor hallucis longus, and flexor digitorum longus on two of the four days. All interventions were performed at the same time of the day. Rolling interventions were performed from proximal to distal and back on the ankle flexors within 2 seconds. Each set lasted 60 seconds, with three sets performed for 180 seconds [21]. According to Kasahara et al., this intervention is equivalent to a rapid FR approach [21]. Nevertheless, the researchers assert that the pace at which the task is performed does not produce different results [21]. The participants were instructed to avoid FR over the Achilles tendon, popliteal fossa, and the origins of the gastrocnemius tendon [19].

### Participants

A total of sixty subjects who engaged in leisure activities were initially included in the study. However, nine subjects did not finish the intervention and were subsequently excluded. These subjects were excluded due to dropouts and absences. Consequently, the sample comprised fifty-one participants. The participants’ mean age was 18.8 years (SD: 5.64), their mean height was 171 cm (SD: 10 cm), and their mean weight was 67.6 kg (SD: 13.5 kg). The study did not include participants who previously had neuromuscular disease or musculoskeletal injury that affected their lower limbs. Furthermore, we included only amateur sports enthusiasts to make the sample analyses as homogeneous as possible. The STROBE flow chart (Figure 1) was used to ensure the assessment of participants of the study was conducted clearly.

### Measurements

#### Ankle dorsiflexion range of motion (ROM)

A Bluetooth inertial sensor was used to quantify the ROM of the ankle joint (Beyond, Motustech SRL, Guidonia Montecelio, Roma, Italy) [30]. The inertial sensor is capable of sampling at a rate up to 1000Hz. The resolution of the accelerometer ranges from ± 2 G to ± 16 G. The gyroscope has a range of ± 200°/s to ± 2000°/s. The magnetometer has a range of ± 4000 microteslas (μT).

Each participant had an ad hoc strap connected to the dorsum of their foot to secure the device during the measurements [31]. Each participant, who was seated with their knee bent, was instructed to actively perform a maximum dorsiflexion movement of the ankle joint. The dorsiflexion was performed for both feet of all participants.

The data collected by the inertial sensor was transferred wirelessly to the related PC software. The following parameters were considered: a) dorsiflexion ROM (°); b) angular speed (°/s): it is the measure of the average angular speed throughout the full ROM; c) fluency index: it is a quantitative measure that ranges from 0 to 1 and represents the level of fluency during the movement (a higher value approaching 1 indicates a more fluid movement).

### Counter-Movement Jump (CMJ) test

An optical detecting system was used to measure the CMJ performance (Optojumpt Next; Microgate SRL; Bolzano, Italy). The technology enables a measurement of flight duration and contact moments throughout the execution of a sequence of jumps, with an accuracy of 1/1.000 of a second. The optical detecting system consists of a transmitting and a receiving bar. Using this technology, the dedicated software may accurately and instantly collect a range of performance parameters [32].

During the test, participants started by standing erect with their hands on their hips. They quickly bent their knees to around a 90° angle and then jumped as far as they could in the subsequent phase of maximum extension [33]. Participants were instructed to elevate from the ground with fully extended knees and ankles and then land in a fully extended position. Everyone underwent three trials, with a 2-minute interval between jumps. The analysis focused on the best performance recorded.

### Intervention: Foam Rolling and Static Stretching (FR-SS) vs. Static Stretching (SS)

#### SS Intervention

The participants in the SS group engaged in a wall stretch exercise for both legs as part of their SS practice. The ankle flexors were specifically addressed in each stretch, with a duration of three sets lasting 30 seconds each, and a 15-second rest period between sets. To perform the stretch, participants positioned themselves by placing one leg on the edge of a bench, straightening the knee, flexing the ankle upwards, and directing the heel towards the ground. They were permitted to rest on the wall to maintain their equilibrium. This procedure, derived from Škarabot et al., was replicated for each leg [34]. This program was followed by a lengthening of the sole of the foot. This SS exercise involved on stretching the sole of the foot, namely the plantar region. The participants positioned themselves in front of a wall, aligning the toes and the front half of the foot against it, while ensuring that the heel remained in contact with the floor. For a stretching sensation, the participant exerted pressure on the ball of their foot, directing it towards the floor, and maintained this position at the maximum level of tension they could tolerate. This procedure, derived from Konrad et al., was replicated for each foot [35, 36].

### FR Intervention

The participants in the FR-SS added a FR program at the SS. The participants were provided with an explanation of using foam rollers by a sports science researcher. A medium-hard expanded polypropylene foam roller measuring 45 cm in length and 15 cm in diameter was utilized for the gastrocnemius and soleus muscles. A smaller roller (measuring 15 × 5.3 × 5.3 cm and weighing 18 grams) made of expanded polypropylene was used for the foot. To become acquainted with the foam roller, participants engaged in two practice sessions a lot of days before the foam roller intervention. This technique ensured that participants were able to execute the FR intervention at the designated duration, velocity, and location. In line with the study by Kasahara et al. [21], the FR intervention comprised three sets, each lasting 60 seconds, with a 30-second break between sets. During each cycle of FR, there was a sequential rolling motion starting from one end to the other, moving towards the center, completed within a time frame of 2 seconds per cycle. This rolling motion was repeated 30 times in each set, resulting in a total of 90 repetitions. A metronome (Smart Metronome, Metronome Beats for iPhone) was employed to regulate timing. Participants were directed to place as much body weight on the roller as they could handle. A competent trainer supervised all FR interventions.

### Statistical analysis

All data were recorded in an Excel spreadsheet (Microsoft Corporation, Redmond, WA, USA). Statistical analysis was conducted using Jamovi software (version 2.3.21.0). The Shapiro-Wilk test was employed to verify data normality. Consequently, data were reported as means and standard deviations (SD). The student’s t-test was used to analyze the possible differences in anthropometric characteristics. A repeated measures ANOVA was conducted to examine the effects of FR-SS and SS on ankle dorsiflexion ROM and jumping ability. The analysis included two variables: time (T0 vs. T1, as within-subject factor) and intervention (FR-SS vs. SS, as between-subject factor), assessing both main effects and interactions. Effect size (ES) classification was defined as follows: $\eta_{\text{p}}^{2}$ less than 0.01 was considered small; between 0.02 and 0.1 was considered medium; and greater than 0.1 was considered a large effect size [37]. Bonferroni-corrected post-hoc analyses were used to examine statistically significant main effects. The significance level was set at p ≤ 0.05.

### Ethics

The study was carried out in compliance with the principles of the Declaration of Helsinki and was approved by the Bioethics Committee of University of Palermo (n. 94/2022—Prot. 70310). Written informed consent was obtained from all participants before participating in the study.

### RESULTS

**FIG. 2:**
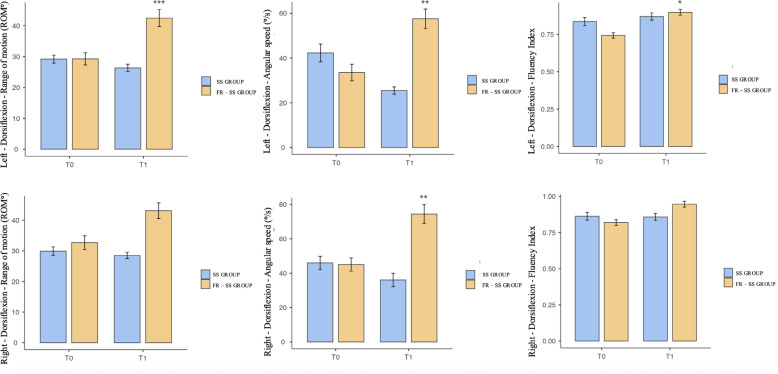
Differences in ankle dorsiflexion before (T0) and after (T1) the interventions for both groups

**FIG. 3:**
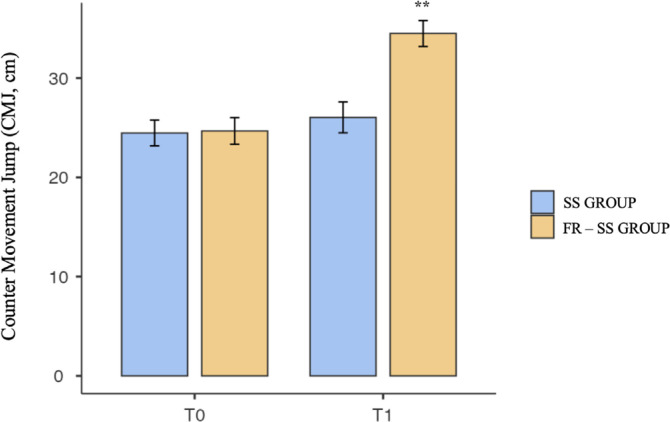
Differences in jumping ability before (T0) and after (T1) the interventions for both groups.

Fifty-one participants completed the intervention and were included in this study. Participants were randomized into two groups: FR-SS, which comprised 22 subjects (15 male and 7 female), and the SS group, which comprised 29 subjects (20 male and 9 female). A retrospective sample size power was performed for a repeated-measures ANOVA using G*Power 3.1 software. The analysis, with an effect size of 0.25 and a type I error rate of 0.05, achieved a power of 0.93. The anthropometric characteristics for each group are summarized in Table 1. Our results showed significant differences between T0 and T1 in the FR-SS group. The repeated-measures ANOVA showed significant group-by-time interaction effects (F = 14.44; p < 0.001, η^2^p = 0.128) and between-subject effects (F = 53.5; p < 0.001, η^2^p = 0.353). Post hoc test results indicated that the variables improved in the FR-SS group compared to the SS group. A detailed Bonferroni analysis can be found in Table 2 and Figure 2. Figure 3 shows the difference in performance on the CMJ before and after the interventions. The FR-SS group showed a significant difference between T0 and T1 (CMJ: p < 0.01; Left–Dorsiflexion ROM: p < 0.001; Angular speed: p < 0.01; Fluency Index: p < 0.05; Right–Dorsiflexion ROM: Angular speed: p < 0.01).

**TABLE 1:** Anthropometric characteristics of each group and results of the student’s t-test for each variable.

	FR-SS Group	SS Group	p*
Participants (n)	22	29	
Age (years)	19.3 ± 6.87	18.5 ± 4.60	ns
Height (cm)	171 ± 7.91	171 ± 11.5	ns
Weight (kg)	66.7 ± 9.52	68.3 ± 16	ns

Legend. ns = not significant;

* p < 0.05;

** p < 0.01; *** p < 0.001

**TABLE 2:** Measures before (T0) and after (T1) the interventions.

		FR–SS GROUP (n = 22)		SS GROUP (n = 29)	

		T0	T1	T0	T1
CMJ (cm)		24.7 ± 6.32	34.5 ± 6.13**	24.5 ± 6.99	26 ± 8.39
Left – Dorsiflexion	ROM (°)	29.3 ± 9.29	42.5 ± 12.8***	29.2 ± 6.91	26.4 ± 6.27
	Angular speed (°/s)	33.5 ± 17.3	57.5 ± 20.6**	42.3 ± 21.5	25.5 ± 8.89
	Fluency Index	0.743 ± 0.08	0.897 ± 0.09*	0.835 ± 0.147	0.869 ± 0.129

Right – Dorsiflexion	ROM (°)	32.7 ± 10.6	43.1 ± 12.1	29.9 ± 7.54	28.5 ± 5.68
	Angular speed (°/s)	45 ± 18	74.4 ± 25.7**	46 ± 20.6	36.1 ± 21
	Fluency Index	0.820 ± 0.09	0.946 ± 0.09	0.862 ± 0.146	0.858 ± 0.127

Legend. ns = not significant;

* p < 0.05;

** p < 0.01;

*** p < 0.001

### DISCUSSION

This study aimed to assess the impact of a 5-week intervention that combined foam rolling (FR) and static stretching (SS) on ankle dorsiflexion range of motion (ROM) and jumping ability.

A 180-second intervention on three sets of FR demonstrated beneficial effects on dorsiflexion parameters in both ankles, but not all enhancements were statistically significant. All parameters of the left ankle showed significant improvement. The right ankle followed a similar trend, but only angular speed showed a statistically significant difference. These effects are in line with the results obtained by Kasahara et al. [21]. The authors showed enhancements in the knee in flexion ROM, maximal voluntary concentric contractions, pain pressure threshold, and tissue stiffness following a comparable treatment program. Furthermore, our data show an enhancement in the angular speed of dorsiflexion in both ankles, suggesting not just a progression in the ROM but also an improvement in the movement patterns of dorsiflexion. Existing studies in the literature have highlighted the significance of ankle angular velocity as a measure of its functional integrity. The study conducted by Srivastava in 2024 demonstrated that ankle angular velocity and acceleration can serve as indicators of compromised dorsiflexion function during walking [38]. Moreover, the authors demonstrated the validity and user-friendliness of accelerometers as efficient instruments for quantifying ankle dorsiflexion. However, further research is necessary to confirm the application.

The literature suggests and demonstrates associations between the ROM in ankle dorsiflexion and the height of CMJ [39, 40]. The results of our study support the conclusion that an enhancement in dorsiflexion is associated with an increase in CMJ height. In addition, the effects were only observed in the group that received the FR intervention together with SS and not in the group that solely followed the SS program. In 2023, Li FY et al. demonstrated that SS reduced explosive performance [41]. However, conclusions on the effects of FR on the CMJ are unclear. The immediate impact of FR on the CMJ has been investigated to evaluate fatigue and performance, particularly in the vertical leap. While several studies have shown that FR for 30 to 60 seconds can result in immediate enhancements in CMJ [42, 43], others have reported no changes following a similar program [44, 45].

To our knowledge, only one study has investigated the long-term effects of a FR intervention [22]. Hodgson et al. suggested that the acute benefits generated by rolling may be transient [22]. However, to the best of our knowledge, this is the first study to examine the cumulative effect of SS combined with a FR intervention. Some studies suggest that the immediate effects of FR may be attributed to enhanced local blood circulation, which is supported by the dilatation of blood vessels and the replenishment of creatine phosphate [46, 47]. On the contrary, supported by the literature [48, 49], we hypothesize that this combined program influenced these results. Some studies have combined FR programs with other techniques that act on muscle tension and lengthening, obtaining favourable variations in the delivery of muscle strength [48, 49]. Muscle power results from various muscle processes, including recruiting muscle fibres. The effects of myofascial release might explain this phenomenon, as this intervention could enhance fibre recruitment patterns through neural stimulation and the breaking down of adhesions, thereby improving muscle power [43, 49, 50]. The combined program is likely to improve resting muscle length and joint angle, enhancing dorsiflexion ROM and thereby increasing peak force [49, 51].

This study is not without limitations. First, the accuracy of the inertial sensor used, although the validity and reliability of instruments with similar technology have been widely demonstrated [31]. Furthermore, its earlier version was documented in the literature [52].

### Practical implications

Practical applications are diverse. Dorsiflexion impacts ankle stability and the risk of injuries like sprains. Additionally, if the effects on force output are validated by further research, this could have implications for both injury recovery and performance enhancement. Provided that additional research validates these results, foam rolling has the potential to be used extensively in sports, functional rehabilitation, and injury prevention. In functional rehabilitation, foam rolling may be used as a non-invasive method to restore patient mobility and strength. Foam rolling could also play a valuable role in fall prevention among the elderly. Regular use of foam rolling could also complement other fall prevention strategies, such as strength training and balance exercises. The potential of foam rolling to improve health and performance underscores the necessity for further studies to validate these findings.

## CONCLUSIONS

In conclusion, our findings indicated that a concurrent 5-week program including FR and SS resulted in enhancements in ankle ROM and angular velocity when compared to SS alone. Furthermore, notable improvements were registered in the performance of countermovement jumps.
